# Suppression of neuroinflammation in forebrain-specific Cdk5 conditional knockout mice by PPARγ agonist improves neuronal loss and early lethality

**DOI:** 10.1186/1742-2094-11-28

**Published:** 2014-02-05

**Authors:** Elias Utreras, Ryusuke Hamada, Michaela Prochazkova, Anita Terse, Satoru Takahashi, Toshio Ohshima, Ashok B Kulkarni

**Affiliations:** 1 Functional Genomics Section, Laboratory of Cell and Developmental Biology, National Institute of Dental and Craniofacial Research, National Institutes of Health, Bethesda, MD, USA; 2 Department of Life Science and Medical Bioscience, Waseda University, Tokyo, Japan; 3 Department of Pediatrics, Asahikawa Medical College, Asahikawa, Hokkaido, Japan; 4 Current address: Biology Department, Faculty of Science, University of Chile, Santiago, Chile

**Keywords:** Neuroinflammation, Cdk5, Pioglitazone, tPA, Cdk5 conditional knockout mice

## Abstract

**Background:**

Cyclin-dependent kinase 5 (Cdk5) is essential for brain development and function, and its deregulated expression is implicated in some of neurodegenerative diseases. We reported earlier that the forebrain-specific Cdk5 conditional knockout (cKO) mice displayed an early lethality associated with neuroinflammation, increased expression of the neuronal tissue-type plasminogen activator (tPA), and neuronal migration defects.

**Methods:**

In order to suppress neuroinflammation in the cKO mice, we first treated these mice with pioglitazone, a PPARγ agonist, and analyzed its effects on neuronal loss and longevity. In a second approach, to delineate the precise role of tPA in neuroinflammation in these mice, we generated Cdk5 cKO; tPA double knockout (dKO) mice.

**Results:**

We found that pioglitazone treatment significantly reduced astrogliosis, microgliosis, neuronal loss and behavioral deficit in Cdk5 cKO mice. Interestingly, the dKO mice displayed a partial reversal in astrogliosis, but they still died at early age, suggesting that the increased expression of tPA in the cKO mice does not contribute significantly to the pathological process leading to neuroinflammation, neuronal loss and early lethality.

**Conclusion:**

The suppression of neuroinflammation in Cdk5 cKO mice ameliorates gliosis and neuronal loss, thus suggesting the potential beneficial effects of the PPARγ agonist pioglitazone for the treatment for neurodegenerative diseases.

## Background

Cyclin-dependent kinase 5 (Cdk5) is an atypical member of the cyclin-dependent kinase family, whose other members are key regulators of the cell cycle [1]. Cdk5 is ubiquitously expressed, and it is mainly active in post-mitotic neurons, where its activators p35 and p39 are predominantly expressed [2,3]. Cdk5 is a proline-directed serine/threonine kinase that phosphorylates a consensus sequence of (S/T)PX(K/H/R) in many target proteins [4]. Cdk5 phosphorylates many cytoskeletal elements, signaling molecules, ions channels, nociceptors and regulatory proteins that participate in the normal function of the brain and also during neurodegenerative disorders [1,3,5,6]. A detailed analysis of Cdk5 knockout mice, which display perinatal lethality and extensive neuronal migration defects, revealed that Cdk5 serves as key regulator of neuronal migration, neurite outgrowth, axonal pathfinding and dendrite development [7,8]. To circumvent the perinatal lethality of Cdk5 knockout mice, we previously generated Cdk5 conditional knockout (cKO) mice that lacked Cdk5 expression in the forebrain [9,10]. At three months of age, these mice displayed an early lethality associated with neuroinflammation, increased expression of the neuronal tissue-type plasminogen activator (tPA) and tumor necrosis factor-α (TNF-α) [10]. Since PPARγ agonist pioglitazone has been reported to effectively suppress neuroinflammation in an Alzheimer’s disease (AD) mouse model [11] and it also had beneficial effects in mouse models of multiple sclerosis (ALS) [12-15] and Parkinson’s disease (PD) [16], we treated cKO mice with pioglitazone, and analyzed its effects on longevity, neuronal loss, and studied the timing of activation of astrocytes and microglial cells by using immunofluorescence against anti-GFAP and Iba1, respectively. In a second approach, we generated Cdk5 and tPA double knockout (dKO) mice to observe effects of the loss of tPA expression on neuroinflammation, astrogliosis and longevity. Our studies revealed the beneficial effects of pioglitazone treatment on the cKO mice. However, the loss of tPA expression in the forebrain of the cKO mice did not ameliorate neuroinflammation and early lethality in these mice.

## Methods

### Animals and experimental protocols

Cdk5^f/f^ mice [9] were crossed with CamKII-Cre^+^ transgenic mice [17] to delete Cdk5, specifically in forebrain adult neurons and these mice were named Cdk5 cKO1 mice [10]. To avoid possible effects of Cre-mediated deletion of the noncoding sequence of flanking genes located in the proximity of *Cdk5* gene, *Accn3* and *Slc4a2*, in Cdk5 cKO1 mice, we crossed Cdk5^f/-^, Cdk5^+/-^[7], and CamKII-Cre^+^ mice to generate Cdk5^f/-^; CamKII-Cre^+^ mice, named Cdk5 cKO2 mice [10]. Additionally, we crossed tPA knockout mice [18] with Cdk5 cKO2 mice to generate double knockout mice: Cdk5 cKO; tPA KO named dKO mice (Additional file 1: Figure S1). The genotype of each mouse was determined by the PCR from DNA obtained from tail biopsies. These studies were performed in compliance with the National Institutes of Health Guidelines on the Care and Use of Laboratory and Experimental Animals. All experimental procedures were approved by the Animal Care and Use Committee of the National Institute of Dental and Craniofacial Research, NIH and the Waseda University.

### Antibodies and materials

Anti-NeuN mouse monoclonal antibody (clone A60, 1:500, Chemicon, Temecula, CA, USA), anti-GFAP rabbit polyclonal antibody (1:500, Dako, Carpinteria, CA, USA), and anti-Iba1 rabbit polyclonal antibody (1:500, Wako, Richmond, VA, USA), and anti-Cdk5 (308-CDK5 Phosphosolutions, Aurora, CO, USA) were used for immunohistochemical analysis of Cdk5 cKO and control mice. Anti-Cdk5 (C8 and J3), anti-p35 (C19), and secondary antibodies (HRP-conjugated goat anti-mouse, anti-rabbit antibodies) from Santa Cruz Biotechnology, Inc. (Santa Cruz, CA, USA), GFAP antibody from Cell Signaling Technology (Beverly, MA, USA), anti-NeuN antibody from Millipore (Temecula, CA, USA), histone H1 and α-tubulin antibody from Sigma (St. Louis, MO, USA) were used for the Western blot analysis of Cdk5 cKO2 and control mice.

### PPARγ agonist pioglitazone treatment

Pioglitazone hydrochloride was obtained from TOCRIS Bioscience (Bristol, UK). The mice were fed rodent chow *ad libitum* that was supplemented with 350 ppm pioglitazone hydrochloride from P30. The final dosage of the drug was calculated to 58.3 mg/kg/day of pioglitazone based on an average daily food consumption of 5 g of chow per mouse.

### Histological analyses

After intraperitoneal injections of avertin (250 mg/kg, Sigma, St. Louis, MO, USA) mice were transcardially perfused with freshly prepared 4% paraformaldehyde in 0.1 mol/L cacodylate buffers, pH 7.4 and brains were removed and embedded in a gelatin matrix (NeuroScience Associates, Knoxville, TN, USA). Free-floating sections (35 μm in thickness) were cut on a microtome and processed for staining. Histological analyses with cresyl violet (Nissl stain, Molecular Probe, Eugene, OR, USA), amino-cupric staining, and immunohistochemistry against anti-NeuN (Chemicon, Temecula, CA, USA) and anti-GFAP (DAKO, Glostrup, Denmark) were performed as previously described [10]. The staining specificity of these antibodies was assessed by omission of the primary antibodies.

### Immunohistochemistry

Cdk5 cKO1 and control mice were perfusion-fixed with 4% paraformaldehyde; thereafter, their brains were removed from their skulls and immersed in the same fixative, which was done overnight at 4°C. The brains were then equilibrated in 20% sucrose and embedded in an optimal cutting temperature (OCT) compound. Cryosections of 14-μm thickness were cut with a frozen microtome. The immunostaining of sections was performed as previously described [8]. Briefly, after the blocking of a non-specific binding of antibodies, the sections were incubated with the primary antibodies, which were diluted in PBS/0.01% Triton X-100, overnight at 4°C. They were then washed three times with PBS and incubated with Alexa-Fluor 488 (1:1,000) or Alexa-Fluor 568 (1:1,000) (Molecular Probes, Eugene, OR, USA) secondary antibodies for one hour. After three further washes with PBS, the sections were embedded in Vectashield mounting media (Vector, Burlingame, CA, USA). Images were obtained by a CCD camera (DP70, Olympus).

### Cell count

Neurons, activated astrocytes and microglia were counted in coronal brain sections. All labeled cells within three squares of 0.15 mm^2^ each were counted in coronal sections using a BX-51 Olympus (Tokyo, Japan) microscope (200× magnification: 10× ocular and 20× objective) as described [19]. Six sections from each brain were analyzed and averaged. The mean value from the combined data of at least three animals per time point was calculated. To exclude false-positives, only GFAP- or Iba1-positive signals merged with nuclear staining by diamidino-2-phenylindole (DAPI) staining were counted.

### Neuronal size

To evaluate neuronal size, we stained coronal sections from these mice with NeuroTrace™ 530/615 (red fluorescent Nissl stain from Molecular Probe, Eugene, OR, USA) and DAPI. Images were obtained by a confocal microscope (FV1000, Olympus, Tokyo, Japan). Sizes of CA1 pyramidal neurons (more than 50 neurons from three mice) were measured by Image-J.

### Western blot analysis

Protein extracts were obtained from brain cortex of control, Cdk5 cKO2, and dKO mice at P17 in T-PER buffer (Pierce, Rockford, IL, USA) with protease inhibitor cocktail tablets and phosphatase inhibitor cocktail tablets, PhosSTOP (Roche Diagnostic, Indianapolis, IN, USA), and Western blot analyses were performed as previously reported [20]. Protein concentration of the supernatant was determined using the Bradford Protein Assay (Bio-Rad, Hercules, CA, USA). Proteins were separated in 4 to 12% or 3 to 8% SDS-PAGE gels and transferred to nitrocellulose membranes (Invitrogen, Carlsbad, CA, USA). The membranes were soaked in a blocking buffer (5% nonfat dry milk in PBS with 0.05% Tween-20 (PBST)) for one hour at room temperature, and then incubated overnight at 4°C, with the appropriate primary antibody diluted in the blocking buffer. The membranes were washed in PBST and incubated for one hour at room temperature with the secondary antibodies diluted in blocking buffer. Immunoreactivity was detected by SuperSignal West Pico or Dura Chemiluminescent Substrate (Thermo Scientific, Rockford, IL, USA). Membranes were stripped for 15 minutes at room temperature with Re-blot Plus Strong Solution (Chemicon, Temecula, CA, USA). They were retested with α-tubulin antibodies to normalize for protein loading. The optical densities of the bands were quantified using an image analysis system with Scion Image Alpha 4.0.3.2 software (Scion Corporation, Frederick, MD, USA).

### Cdk5 kinase activity assay

As reported earlier, 300 μg of protein from brain cortex of 17-day-old control, Cdk5 cKO2, and dKO mice, were dissolved in T-PER buffer, and they were immunoprecipitated using 4 μg of anti-Cdk5 (C8) antibody (Santa Cruz Biotechnology, Inc., Santa Cruz, CA, USA) [21]. Immunoprecipitated proteins (IP) were washed three times in cold PBS, and twice in kinase buffer (20 mM Tris HCl (pH 7.4), 10 mM MgCl_2_, 1 mM EDTA). Then, the IP was mixed with the kinase assay mixture (100 mM Tris HCl (pH 7.4), 50 mM MgCl_2_, 5 mM EDTA, and 5 mM DTT) plus 5 units of (γP^32^)-ATP, and using 5 μg of histone H1 as a substrate. The kinase assays were carried out for 30 minutes at 30°C, and the kinase activity reaction was stopped by adding 5× sodium dodecyl sulfate sample buffer and boiling for ten minutes at 70°C. Kinase reaction was electrophoresed on a 4 to 20% polyacrylamide gel, and then gels were exposed to X-ray films, for 1 to 3 hours at -80°C. The incorporation of P^32^ to histone H1 was quantified to measure band intensity using Scion Image Alpha 4.0.3.2 software (Scion Corporation, Frederick, MD, USA).

### RNA isolation and real-time PCR

Total RNAs were obtained from brain cortex of 17-day-old control, Cdk5 cKO2, and dKO mice. RNA was isolated from tissue using TRIzol® reagent (Invitrogen, Carlsbad, CA, USA), according to the manufacturer’s instructions. Following TURBO DNA-free (Ambion, Austin, TX, USA) digestion of the total RNA sample, oligo (dT)-primed synthesis of cDNA from 3 μg of total RNA was made using SuperScript™ III Reverse Transcriptase (Invitrogen, Carlsbad, CA, USA) to remove contaminated genomic DNA. For detection of CD68, TNF-α, interleukin 6 (IL-6), interferon-γ (IFN-γ), interleukin 1β (IL-1β), transforming growth factor β1 (TGF-β1), oncostatin M (OSM) and leukemia inhibitory factor (LIF) mRNA, we used real-time PCR, and the following reaction mixture was used for these PCR samples: 1xIQ™ Sybr®Green Super Mix (Bio-Rad, Hercules, CA, USA), 100 to 200 nM of each primer and 1 μl of cDNA. cDNA was amplified and analyzed in triplicate using Opticon Monitor Chromo 4 (Bio-Rad, Hercules, CA, USA). The following primers were used to amplify and measure the amount of mouse mRNA by real-time PCR: CD68 sense 5′-ACC GCC ATG TAG TCC AGG TA-3′ and antisense 5′-ATC CCC ACC TGT CTC TCT CA-3′; Cdk5 sense 5′-GGC TAA AAA CCG GGA AAC TC-3′ and Cdk5 antisense 5′-CCA TTG CAG CTG TCG AAA TA-3′; TNF-α sense 5′-GAT CTC AAA GAC AAC CAA CTA GT-3′ and antisense 5′-CTC CAG CTG GAA GAC TCC TCC CAG -3′; IL-6 sense 5′-CTG CAA GAG ACT TCC ATC CAG TT -3′ and antisense 5′-GAA GTA GGG AAG GCC GTG G -3′; IFN-γ sense 5′-TCA AGT GGC ATA GAT GTG GAA GAA-3′ and antisense 5′-TGG CTC TGC AGG ATT TTC ATG-3′; IL-1β sense 5′-CAA CCA ACA AGT GAT ATT CTC CAT G-3′ and antisense 5′-GAT CCA CAC TCT CCA GCT GCA-3′, TGF-β1 sense 5′-GCA GTG GCT GAA CCA AGG AG-3′ and antisense 5′–CCC GAC GTT TGG GGC TGA TC-3′; OSM sense 5′-TGT GGC TTT CTC TGG GGA TAC-3′ and antisense 5′-GAA GGT CTG ATT TTG CGG GAT-3′; LIF sense 5′-ACG GCA ACC TCA TGA ACC A-3′ and antisense 5′-GGA AAC GGC TCC CCT TGA-3′. The gene expression level was normalized against S29 expression using S29 sense 5′-GGA GTC ACC CAC GGA AGT TCG G-3′ and antisense 5′-GGA AGC ACT GGC GGC ACA TG-3′. Real-time PCR reactions were run in triplicate and repeated three times.

### Statistical analysis

All of the experiments were performed a minimum of three times. The statistical evaluation was done with GraphPad Prism software, version 4.0 (GraphPad, San Diego, CA, USA). The significant differences between the experiments were assessed by univariate ANOVA (more than two groups) or unpaired *t*-tests (two groups). ANOVA was followed by *t*-tests using a Bonferroni α-correction or Dunett’s test, where α was set to 0.05.

## Results

### Inflammatory reactions precede neuronal loss and early lethality in Cdk5 cKO mice

As reported earlier, we generated two lines of Cdk5 conditional knockout mice: Cdk5 cKO1 (Cdk5^f/f^; CamKII-Cre^+^) and Cdk5 cKO2 (Cdk5^f/-^, CamKII-Cre^+^) mice [10] (see, Additional file 1: Figure S1). Both Cdk5 cKO1 and cKO2 mice were born with an expected Mendelian frequency. As described earlier [10] 20% to 30% of Cdk5 cKO1 mice lived for more than three months; however 95% of the Cdk5 cKO2 mice died within one month after birth. We had earlier observed that the neuroinflammation and neuronal loss occurred at P90 but not at P21 in Cdk5 cKO1 mice as compared to Cdk5^f/f^ controls [10]. To define the precise timing of the onset of this neuroinflammation, we analyzed the inflammatory reactions in the brain of Cdk5 cKO1 mice by immunohistochemical staining. NeuN, GFAP and Iba1 were used as markers for neurons, activated astrocytes and microglia, respectively. At P21, we did not notice any increase in the GFAP-positive or Iba1-positive cells (Additional file 2: Figure S2A-E). At P30, we observed increased GFAP-positive cells and Iba1-positive cells in the brain cortex of Cdk5 cKO1 mice (Additional file 2: Figure S2F-J); however, there was no apparent decrease in the NeuN-positive cells, thus indicating that neuronal loss did not occur at this age. Moreover, we evaluated whether the deficient of Cdk5 in mice produced pathological changes such as degenerative neurons or change in neuron size. We analyzed different areas of control and Cdk5 cKO1 mice at P30 and we performed amino-cupric silver and Nissl stain to evaluate those parameters. We found an increased number of amino-cupric silver positive cells in both cerebral cortex and striatum of Cdk5 cKO1 as compared with control mice (Additional file 3: Figure S3A). At this stage, no difference in neuronal size was found in CA1 pyramidal neurons between Cdk5 cKO1 and control littermates (Additional file 3: Figure S3B).

### Pioglitazone, PPARγ agonist, treatment improved neuronal loss in Cdk5 cKO1 mice

**Figure 1:**
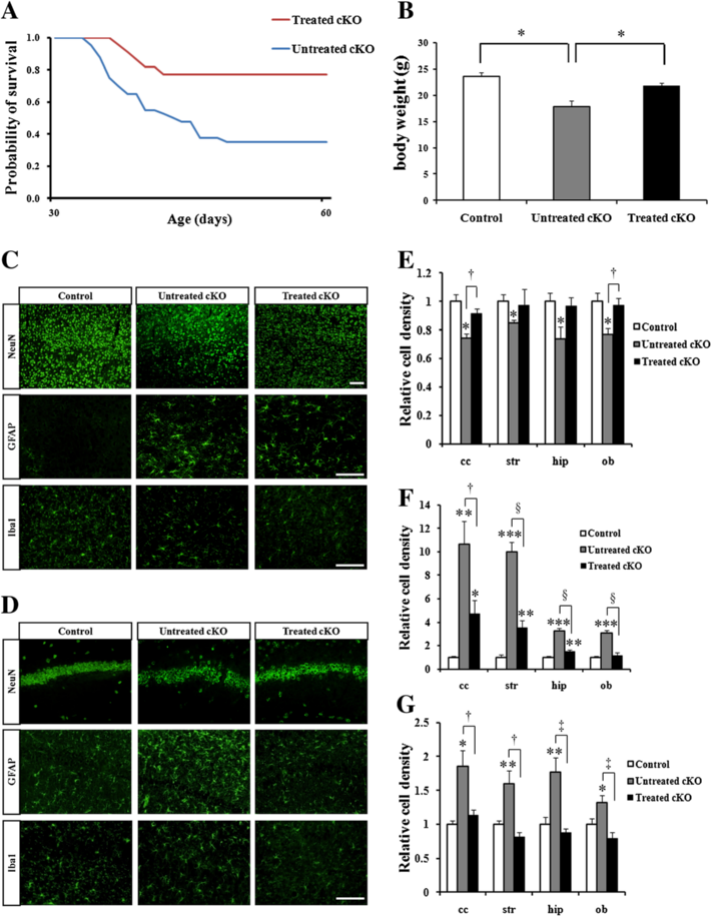
**PPARγ agonist pioglitazone suppressed inflammation and partially reversed neuronal loss in Cdk5 cKO1 mice. (A)** Survival curves for untreated and treated Cdk5 cKO1 mice from P30 to P60. Survival rate for treated mice (n = 16) is better than that of untreated mice (n = 13). **(B)** Average body weights of untreated (n = 3) and treated (n = 3) male Cdk5 cKO1 mice and littermate male Cdk5^f/f^ (control) mice (n = 3) at P60. *P < 0.05. **(C)**, **(D)** Immunohistochemical (IHC) analyses of control, untreated, and treated Cdk5 cKO1 mice in cerebral cortex **(C)** and hippocampus **(D)** at P60. Coronal sections from these mice were stained with antibodies for NeuN, GFAP and Iba1. Scale bar = 100 μm. **(E-G)** Bar graphs indicate average cell densities of neurons **(E)**, activated astrocytes **(F)** and microglia cells **(G)** in control, untreated and treated Cdk5 cKO1 mice at P60. ***P < 0.001 between control versus Cdk5 cKO1. †*P* < 0.05; ‡*P* < 0.01; §*P* < 0.001 between no treated versus treated (*t*-test).

Because we observed neuroinflammation in the Cdk5 cKO1 cortex at P30, we hypothesized that the inflammatory reactions were the cause of neuronal loss [10]. To test this hypothesis, we treated the Cdk5 cKO1 mice with the PPARγ agonist pioglitazone that suppressed inflammation in the brain of an AD mouse model [11]. We started the pioglitazone treatment at P30 and analyzed the results of this treatment at P60 and P90. We compared the survival of the treated and untreated Cdk5 cKO1 mice. At P60, 76% of the Cdk5 cKO1 mice (16/21) survived as compared to 33% of the untreated mice (13/39) (*P* < 0.01; *P* = 0.0080 in Log-rank test and *P* = 0.0086 in Wilcoxon test) (Figure 1A). At P90, a similar higher survival rate was observed in the treated Cdk5 cKO1 mice (Additional file 4: Figure S4A). We next compared the body weight gain in the male mice from both the groups. At P60, untreated Cdk5 cKO1 mice showed reduced body weight whereas the treated Cdk5 cKO1 mice recovered from the body weight loss (control = 23.62 ± 0.68 g, untreated Cdk5 cKO1 mice = 17.79 ± 1.11 g, and treated Cdk5 cKO1 mice = 21.79 ± 0.53 g) (Figure 1B). Improved body weight gain was also observed in the treated Cdk5 cKO1 mice at P90 (Additional file 4: Figure S4B). Since we had earlier observed neuronal loss in the olfactory bulb, brain cortex, striatum and hippocampus of Cdk5 cKO1 mice at P90 [10], we analyzed cell densities of neurons, reactive astrocytes and microglia by using NeuN, GFAP, and Iba1 markers, respectively. At P60, we observed significant neuronal loss in all regions in the untreated Cdk5 cKO1 mice, whereas neuronal loss was significantly reversed in the treated Cdk5 cKO1 mice (Figure 1C-E). We also found a decrease in the number of GFAP-positive astrocytes and Iba1-positive microglia in the treated Cdk5 cKO1 mice, as compared to the untreated mice (Figure 1C-G). Improvement in neuronal loss and suppressed neuroinflammation were also found in the treated Cdk5 cKO1 mice at P90 (Additional file 4: Figure S4C-G). The data indicate that pioglitazone suppressed neuroinflammation and partially rescued neuronal loss in Cdk5 cKO1 mice, suggesting that neuroinflammation caused neuronal loss in the Cdk5 cKO1 mouse brain. As described previously [10], untreated Cdk5 cKO1 mice showed retraction of their limbs toward their trunks when suspended by their tail. Therefore, we analyzed whether pioglitazone treatment can improve the neurological deficit observed in Cdk5 cKO1 mice. We found that this phenotype was partially improved in treated Cdk5 cKO1 as compared with untreated Cdk5 cKO1 or control mice at P60 (Additional file 5: Movie S1).

### Decreased Cdk5 and p35 protein expression and Cdk5 kinase activity in brain of Cdk5 cKO2 and dKO mice

**Figure 2:**
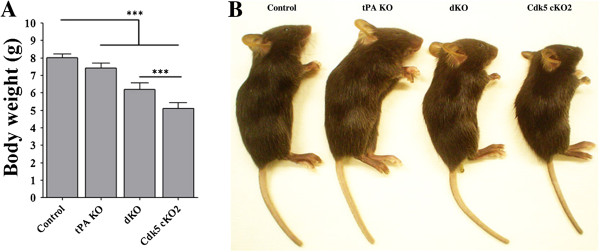
**Generation of Cdk5 cKO2; tPA KO (dKO) mice. (A)** Body weights of 17-day-old mice of four different genotypes. **(B)** Representative images of control, tPA KO, Cdk5 cKO2 and dKO mice. All data are presented as the mean and SEM (n = 6 to 12 mice per group), ***P < 0.001 (*t*-test).

To generate Cdk5 cKO; tPA KO mice (dKO), we crossed Cdk5 cKO2 (Cdk5^f/-^; CamKII-Cre^+^) with tPA knockout mice (tPA^-/-^) (Additional file 1: Figure S1). Both Cdk5 cKO2 and dKO mice were born with an expected Mendelian frequency. Both Cdk5 cKO2 and dKO mice were indistinguishable from the control littermates at birth although some of the Cdk5 cKO2 and dKO mice were born dead. Surprisingly, we observed that nearly 95% of dKO mice died within a week after weaning (P21), similar to the trend we observed in Cdk5 cKO2 [10]. We measured their body weights at P17 and found a significant decrease in body weight gain in the Cdk5 cKO2 and dKO mice as compared to the control mice. Interestingly, body weights of the dKO mice were significantly higher in comparison to the Cdk5 cKO2 mice suggesting a partial rescue in body weight loss in the dKO mice (Figure 2A and B). On the other hand, tPA KO mice did not show any changes in survival rate and body weight gain in comparison with the control mice (data not shown).

**Figure 3:**
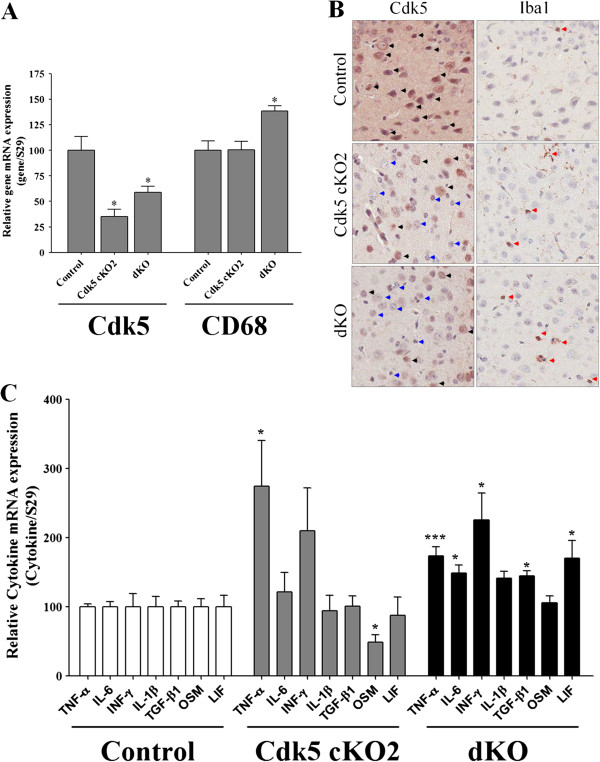
**Increased microgliosis and pro-inflammatory cytokines in the brain of dKO mice. (A)** qPCR analysis of Cdk5 and CD68 mRNA levels (a marker of activated microglia) normalized against S29. Total RNA was obtained from brain cortex of control, Cdk5 cKO2 and dKO mice at P17. There was a decreased Cdk5 mRNA levels on Cdk5 cKO2 and dKO mice. In contrast, CD68 was increased only on dKO mice as compared with control mice. **(B)** Right panel shows a representative IHC against Cdk5 and left panel an IHC against Iba1 of cortex coronal section from control, Cdk5 cKO2 and dKO mice at P17. Decreased Cdk5 expression was observed in the cortex of Cdk5 cKO2 and dKO mice (Blue head arrows as compared with black head arrows). IHC Iba1 shows that Iba1 positive cells are few in the cortex of the control mice as compared with Cdk5 cKO2 and dKO mice (red head arrows). Also, Iba1 positive cells are more apparent on dKO as compared with Cdk5 cKO2 mice. **(C)** Real-time PCR analysis of mRNA levels of several pro-inflammatory cytokines normalized against S29. All data are presented as the mean and SEM (n = 3 to 5). *P < 0.05; ***P < 0.001 (*t*-test).

**Figure 4:**
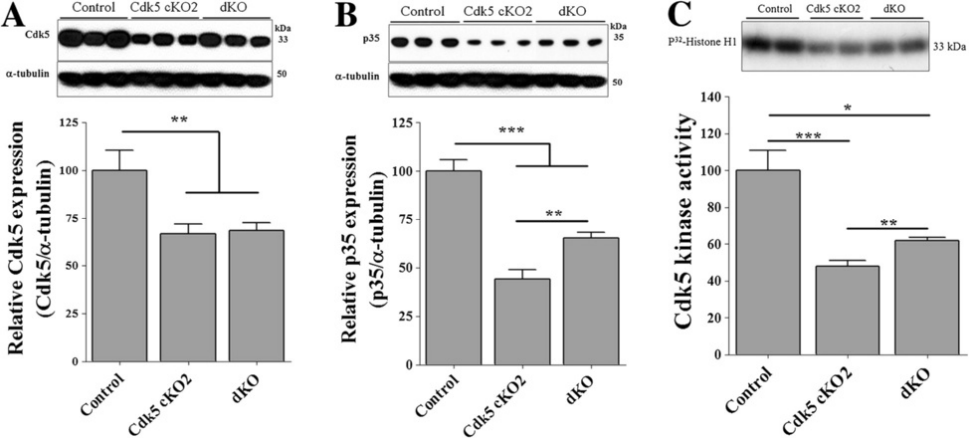
**Decreased Cdk5 and p35 protein expression and Cdk5 activity in the brain of Cdk5 cKO2 and dKO mice. (A)** Representative Western blot analysis showing Cdk5 protein levels in brain cortex of control, Cdk5 cKO2 and dKO mice at P17. Lower panel showed a quantification of Western blots for Cdk5. α-tubulin was used as loading control. **(B)** Representative Western blot analysis showing p35 protein levels in brain cortex of control, Cdk5 cKO2 and dKO mice at P17. Lower panel showed a quantification of Western blots of p35. α-tubulin was used as loading control. **(C)** Cdk5 kinase activity was measured from immunoprecipitates of proteins obtained from brain cortex of control, Cdk5 cKO2 and dKO mice at P17. All data are presented as the mean and SEM (n = 3 to 12). *P < 0.05, **P < 0.01, ***P < 0.001 (*t*-test).

We analyzed the Cdk5 mRNA levels in the brain cortex of P17 mice by using real-time PCR. We found that Cdk5 mRNA levels were significant decreased in both Cdk5 cKO2 and dKO mice as compared to the control mice (Figure 3A). Interestingly, Cdk5 mRNA levels of the dKO mice were significantly higher in comparison to the Cdk5 cKO2 mice, suggesting a partial rescue (Figure 3A). Additionally, we analyzed Cdk5 protein levels in the brain cortex of P17 mice by using Western blot analysis. Cdk5 protein levels were significantly decreased in both Cdk5 cKO2 and dKO mice as compared to the control mice (Figure 4A). Also, we performed IHC against Cdk5 and we found decreased expression of Cdk5 in neurons in the cortex of Cdk5 cKO2 and dKO mice as compared with control mice at P17 (black and blue arrow head Figure 3B left panel) suggesting that remaining Cdk5 positive cells observed in Cdk5 cKO2 and dKO were the glial cells or neuron cells in which Cre-mediated recombination was absent. Additionally, we analyzed the p35 protein levels by Western blot and found a significantly reduced expression in both Cdk5 cKO2 and dKO mice as compared with the control mice. Remarkably, the p35 protein levels were significantly higher in dKO, in comparison with those in Cdk5 cKO2 mice, suggesting again a partial rescue in dKO mice (Figure 4B). Since the p35 expression is a limiting factor for Cdk5 kinase activity [22], we determined whether Cdk5 kinase activity was affected in the brain cortex of mice at P17. We found a significant decrease in Cdk5 kinase activity in cKO2 and dKO mice, as compared with the control mice. Interestingly, Cdk5 kinase activity was significantly higher in dKO in comparison with the Cdk5 cKO2 mice, suggesting a partial rescue in the dKO mice (Figure 4C).

### Increased neuronal loss and reduced astrogliosis in brain of dKO mice

**Figure 5:**
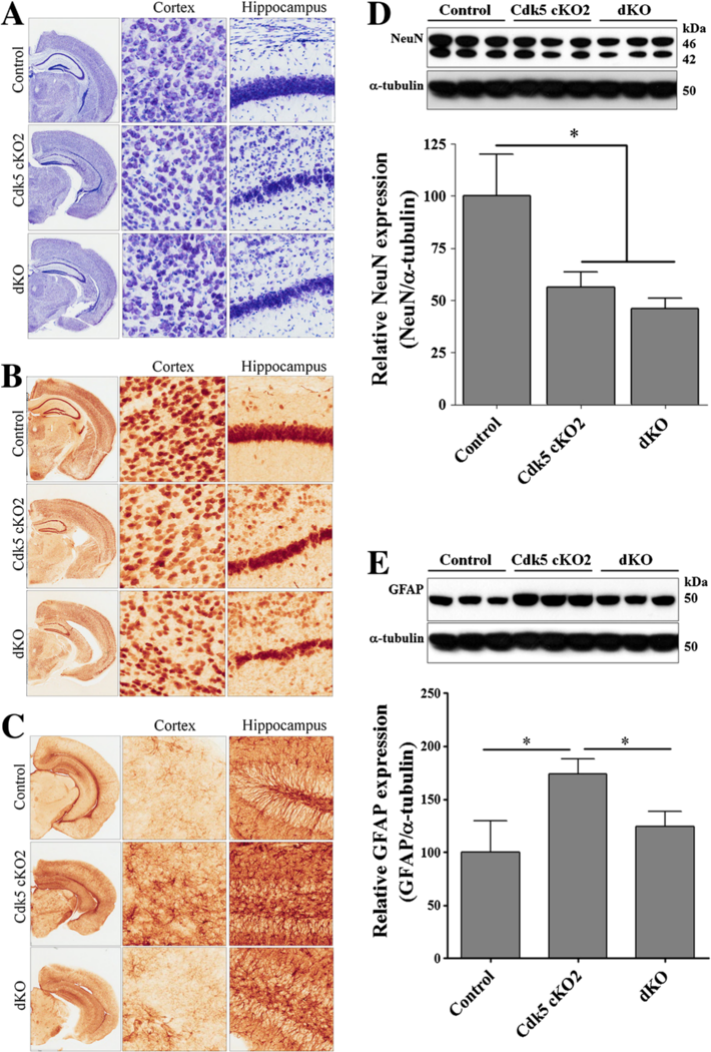
**Increased neuronal loss and partial rescue of astrogliosis in the brain of dKO mice. (A)** Nissl staining of coronal brain sections of control, Cdk5 cKO2 and dKO mice at P17. In higher magnification we observed a decreased of the number of cells on cortex and hippocampus of Cdk5 cKO2 and dKO mice. **(B)** Immunohistochemistry (IHC) against NeuN, a marker of neurons, of coronal brain sections of control, Cdk5 cKO2 and dKO mice at P17. In higher magnification we observed a decreased of the number of NeuN positive cells on cortex and hippocampus of Cdk5 cKO2 and dKO mice. **(C)** IHC against GFAP, a marker of astrogliosis, of coronal brain sections of control, Cdk5 cKO2 and dKO mice at P17. In higher magnification we observed an increased number of GFAP positive cells on cortex and hippocampus of Cdk5 cKO2. In contrast, there were a decreased number of GFAP positive cells on the same areas of brain of dKO mice. **(D)** Representative Western blot analysis showing NeuN protein levels in brain cortex of control, Cdk5 cKO2 and dKO mice at P17. Lower panel showed a quantification of Western blots of NeuN. α-tubulin was used as loading control. **(E)** Representative Western blot analysis showing GFAP protein levels in brain cortex of control, Cdk5 cKO2 and dKO mice at P17. Lower panel showed a quantification of Western blots of GFAP. α-tubulin was used as loading control. All data are presented as the mean and SEM (n = 3 to 6). *P < 0.05 (*t*-test).

We earlier reported that Cdk5 cKO2 mice displayed neuronal loss and increased astrogliosis as determined by the NeuN and GFAP protein levels, respectively [10]. To evaluate whether tPA deficiency in the background of Cdk5 cKO2 mice could improve this phenotype, we analyzed neuronal loss as detected by Nissl staining and NeuN expression, and astrogliosis detected by GFAP protein expression in coronal brain sections of control, Cdk5 cKO2, and dKO mice at P17. We found that both Cdk5 cKO2 and dKO mice showed a decreased Nissl staining (Figure 5A) and also a reduced NeuN expression measured by IHC (Figure 5B) as compared with the control mice. In addition, we found significantly decreased NeuN protein levels in both Cdk5 cKO2 and dKO brain cortex, as compared with the controls at P17 (Figure 5D). We also evaluated astrogliosis by IHC and the Western blot of GFAP in these mice. As reported earlier [10], we found increased GFAP staining by IHC; and a significant increase in GFAP protein expression by Western blot analysis in the brain cortex of Cdk5 cKO2 mice as compared with the control mice. Surprisingly, the levels of GFAP in the dKO mice were reversed to near normal levels (Figure 5C and E) suggesting a partial reversal in the gliosis in these mice.

### Increased microgliosis and pro-inflammatory cytokines in the brain of dKO mice

We had reported earlier increased microglia activation measured by F4/80 marker and CD68 mRNA levels in the forebrain of Cdk5 cKO1 mice at two to three months old [10]. To extend these observations to the Cdk5 cKO2 and dKO mice, we evaluated the CD68 mRNA levels and Iba1 protein expression in the brain cortices of these mice. At P17 the CD68 mRNA levels were similar in both the control and Cdk5 cKO2 mice. However, these levels were significantly increased in dKO mice as compared with the control and Cdk5 cKO2 mice (Figure 3A). Additionally, we evaluated whether Cdk5 deletion in the brain cortex of Cdk5 cKO2 and dKO mice could increase Iba1 positive cells by using IHC (Figure 3B). We found that in the same areas of the cortex of Cdk5 cKO2 mice where Cdk5 was deleted (black and blue head arrow Figure 3B left panel), there was an increase of Iba1 positive cells as compared with control mice (red head arrow, Figure 3B right panel). Remarkably, we found more Iba1 positive cells on cortex of dKO mice as compared with Cdk5 cKO2 mice suggesting increased microglial activation in the dKO mice (Figure 3B right panel). As reported earlier, the TNF-α levels were increased in Cdk5 cKO1 mice at two to three months old [10]. To extend these observations to the Cdk5 cKO2 and dKO mice, we evaluated several pro-inflammatory cytokines mRNA levels in the brain cortex of control, Cdk5 cKO2 and dKO mice at P17. We evaluated the expression of pro-inflammatory cytokines such as TNF-α, IL-6, IFN-γ, IL-1β, TGF-β1, OSM, and LIF in the brain cortices of mutant and control mice at P17. Real-time PCR analysis with specific primers for all of the indicated cytokines revealed that only TNF-α mRNA levels were significantly increased, and OSM mRNA levels were decreased in Cdk5 cKO2, as compared with the control mice (Figure 3C). Most importantly, we found that mRNA levels of TNF-α, IL-6, IFN-γ, TGF-β1, and LIF were significantly increased in the dKO mice as compared with the control mice (Figure 3C), suggesting that increased glial activation induced higher levels of cytokines in dKO mice.

## Discussion

The conditional deletion of neuronal Cdk5 in the adult forebrain caused activation of astrocytes and microglia and neuroinflammation, and a subsequent neuronal loss and early lethality. In our first approach to suppress neuroinflammation in these mice, treatment with PPARγ agonist pioglitazone resulted in a significant reduction in microgliosis, astrogliosis, and neuronal loss associated with a better survival rate. In the second approach, we evaluated the potential role of tPA in neuroinflammation in Cdk5 cKO mice by generating dKO mice in which the *tPA* gene was deleted. The deletion of tPA in the Cdk5 KO mice caused a partial rescue in astrogliosis, although there was increased microgliosis and inflammatory cytokines levels. Nevertheless, the dKO mice still died at P21 similar to the Cdk5 cKO2 mice, suggesting that tPA does not play a major role in the etiology of neuroinflammation.

Differences in the longevity rate between the Cdk5 cKO1 and cKO2 mice are because the cKO2 mice have significantly reduced Cdk5 protein levels, as compared with Cdk5 cKO1 mice [10]. Cdk5 cKO2 mice have one allele knocked out in all cells of the body whereas another allele is deleted only in adult forebrain neurons. However, in Cdk5 cKO1 mice both alleles are deleted only in adult forebrain neurons, suggesting that expression levels of Cdk5 have a greater impact on the survival rate of these mice. Our results confirm several earlier reports indicating that Cdk5 kinase activity is required for survival of neurons under stressful conditions [23-26]. Cdk5 is known to prevent neuronal apoptosis by negative regulation of JNK [23]. It is also involved in the regulation of Akt activity mediating neuronal survival [24]. Cdk5 also plays critical roles in non-neuronal cells, such as oligodendrocytes and astrocytes. Thus, Cdk5 regulates oligodendrocytes differentiation and myelin repair [27-30]. Moreover, it was shown that Cdk5/p35 is expressed in astrocytes and it regulates the process of elongation of the scratched astrocytes [31]. Here, we found neuroinflammation without neuronal loss at P30 in Cdk5 cKO1 mice. However, we observed pathological changes in neurons by silver staining at P30, indicating that neuroinflammation might trigger neuronal degeneration, which could be contributing to the neuronal loss at late stages. Interestingly, we found an increased astrogliosis and neuronal loss much early in the cerebral cortex of Cdk5 cKO2 (P17) in comparison with Cdk5 cKO1 mice (P30), which suggests that the decreased Cdk5 levels in Cdk5 cKO2 mice accelerate neuroinflammation with a consequent neuronal loss and an early lethality.

PPARγ agonists inhibit the production of many inflammatory cytokines such as TNF-α, IL-1β and IL-6 in several cell types, including monocytes, macrophages and epithelial cells [32]. This inhibitory effect occurred through silencing the action of the transcription factors, NFκB and AP-1, on the promoters of the cytokines and others genes [32-34]. On the other hand, microglia are derived from hematopoietic precursors that enter the developing CNS to become a major population of resident macrophages in brain. PPARγ agonists can potentially control neuroinflammation through their actions on the activated microglia which can produce, similar to peripheral macrophages, pro-inflammatory and neurotoxic factors, including cytokines, reactive oxygen intermediates, and nitric oxide [35]. PPARγ agonist pioglitazone has been reported to have beneficial effects on neurodegenerative disease mouse models [36-38]. Thus, the pioglitazone treatment seems to offer a broad range of potentially protective properties that attenuate the chronic neuroinflammation and oxidative stress that is responsible for the progression of PD [36]. Pioglitazone treatment also has inhibitory effects on STAT3 activation in ALS mouse models [37]. Additionally, it was reported that pioglitazone treatment of amyloid precursor protein/presenilin 1 mice (an AD mouse model) enhanced the microglial uptake of Aβ and spatial cognitive memory improvement [38]. Here, we have found that pioglitazone treatment suppressed neuroinflammation by reducing activation of microglia and astrocytes in Cdk5 cKO1 mice. Indeed, we found that pioglitazone treatment can improve the neurological deficit observed in Cdk5 cKO1 mice. Similarly, the survival rate and body weight gain was significantly increased in the Cdk5 cKO1 mice treated with pioglitazone as compared to the untreated Cdk5 cKO1 mice. These results suggest that a decrease in neuronal Cdk5 expression produces an increased neuroinflammation, resulting in neuronal loss and death.

In addition to the effects of PPARγ agonists on PPARγ activity, it was recently demonstrated that troglitazone, another PPARγ agonist, decreased tau-Thr231 phosphorylation [39]. This repression was brought about by inhibition of the Cdk5 kinase activity caused by an inhibition of proteasomal degradation of p35 in human neuroblastoma SH-SY5Y cells and rat primary cortical neurons in culture [39]. Moreover, altered Cdk5 activity has been associated with many neurodegenerative diseases, which is mainly regulated by the increased proteolysis of p35 into p25, with a subsequent increased Cdk5 kinase activity [40-42]. Therefore, the beneficial effects of PPARγ agonist treatment in neurodegenerative diseases could be due to inhibition of Cdk5 kinase activity and a subsequent reduction in tau phosphorylation. Additionally, it will be of interest to explore potential beneficial effects of these agonists for treating painful disorders since Cdk5 has also been implicated in pain signaling [6].

Two important questions that need to be answered are: How microglia and astrocytes are activated? And which molecules released from neurons are activating microglia and astrocytes in Cdk5 cKO mice? In our previous work, we found increased tPA mRNA levels and tPA activity in Cdk5 cKO1 mice [10] and because neurons are known to release tPA and activate microglia through annexin II [43], we therefore evaluated here whether tPA plays a major role in neuroinflammation and neuronal loss observed on Cdk5 cKO mice. Additionally, it was reported that tPA can exert neurotoxic effects in a catalytic-independent way by activating the ERK1/2 signaling pathway and that this molecular mechanism could be responsible for the neuronal death induced in mouse hippocampal neurons after Aβ treatment [44]. Moreover, the same group also reported that Aβ treatment of tPA deficient glial cells results in a dramatic reduction of inflammation analyzed by TNF-α expression [45]. Here, we generated dKO mice that lack tPA in all cells and Cdk5 in forebrain neurons. In these mice, we found a partial rescue in astrogliosis, but not in microgliosis. We also observed increased CD68 mRNA levels and increased Iba1 positive cells, markers of microgliosis, in dKO mice. In contrast, it was reported that tPA treatment did not increase Iba1 expression on microglia from mouse spinal cord [46], suggesting an unknown mechanism of microglia activation caused by a deficiency of tPA. Finally, we found elevated levels of inflammatory cytokines in dKO mice in comparison with control mice, suggesting that the increased expression of tPA observed in the Cdk5 cKO1 mice does not contribute significantly to the pathological process that leads to neuroinflammation, neuronal loss and early lethality.

## Conclusion

We have demonstrated that tPA does not play a major role in the etiology of neuroinflammation seen in Cdk5 cKO mice; however, the treatment with pioglitazone significantly reduced the microgliosis, astrogliosis, and neuronal loss and improved the neurological deficit in Cdk5 cKO mice suggesting a beneficial effect of this PPARγ agonist in treating neurodegenerative diseases.

## Abbreviations

Aβ: amyloid beta; AD: Alzheimer’s disease; CaMKII: calcium/calmodulin-dependent kinase II; Cdk: cyclin-dependent kinase; cKO: conditional knockout; CNS: central nervous system; DAPI: 4′,6-diamidino-2-phenylindole; dKO: double knockout; EDTA: ethylenediaminetetraacetic acid; GFAP: glial fibrillary acidic protein; HRP: horse radish peroxidase; Iba1: ionized calcium binding adaptor molecule 1; IgG: immunoglobulin G; IHC: Immunohistochemical; IL-1β: interleukin-1 beta; IL-6: interleukin-6; IP: immunoprecipitated proteins; JNK: Jun N-terminal kinase; LIF: leukemia inhibitory factor; NeuN: neuronal nuclei NFκB: nuclear factor kappa B; O.C.T.: optimal cutting temperature; OSM: oncostatin M; PBS: phosphate buffered saline; PCR: polymerase chain reaction; PPAR: peroxisome proliferator-activated receptor; SDS: sodium dodecyl sulfate; STAT3: signal tranducer and activator of transcription 3; TGF-β1: transforming growth factor β1; TNF-α: tumor necrosis factor alpha; tPA: tissue-type plasminogen activator; Triton X-100: polyoxyethylene(10) octyl phenyl ether.

## Competing interests

The authors declare that they have no competing interests.

## Authors' contributions

EU, TO, ABK designed research; EU, RH, MP, AT, ST, TO and ABK performed research; EU, RH, MP, TO, and ABK analyzed data, and EU, TO and ABK wrote the paper. All authors read and approved the final manuscript.

## Supplementary Material

Additional file 1: Figure S1 Generation of Cdk5 cKO2, tPA KO mice. Cdk5 floxed (Cdk5^f/f^) mice were crossed with tPA knockout mice (tPA KO) to generate Cdk5^f/f^; tPA^+/-^ mice. In addition, Cdk5^+/-^ mice [7] were crossed with either CamKIIα-Cre^+ ^[17] and tPA KO mice to generate Cdk5^+/-^; CamKIIα-Cre^+^ and Cdk5^+/-^; tPA KO mice, respectively. Lately, Cdk5^+/-^; CamKIIα-Cre^+^ mice were crossed either with Cdk5^f/f^; tPA^+/-^ and Cdk5^+/-^; tPA KO mice to generate Cdk5^f/-^; CaMKIIα-Cre^+^; tPA^+/-^ and Cdk5^+/-^; CamKIIα-Cre^+^; tPA^+/-^. Finally, these mice were crossed to generate Cdk5^f/-^; CamKIIα-Cre^-^; tPA^+/-^ (control mice), Cdk5^f/-^; CamKIIα-Cre^+^; tPA^+/-^ (Cdk5 cKO2 mice) and Cdk5^f/-^; CamKIIα-Cre^+^; tPA^-/-^ (dKO mice). Click here for file

Additional file 2: Figure S2 Inflammation proceeds neuronal loss in Cdk5cKO mice. (A, B) IHC analysis of coronal brain sections of P21 day-old mice. Sections of cerebral cortex (A) and hippocampus (B) from control and Cdk5 cKO1 mice were stained with antibodies for NeuN, GFAP and Iba1. Scale bar = 100 μm. (C-E) Bar graphs of average cell densities of neuron (C), activated astrocytes (D) and microglia (E) in each brain region of Cdk5 cKO1 mice and littermate controls at P21. (F, G) IHC analysis of coronal brain sections of P30 day-old mice. Sections of cerebral cortex (F) and hippocampus (G) of the control and Cdk5 cKO1 mice were stained with antibodies for NeuN, GFAP and Iba1. Scale bar = 100 μm. (H-J) Bar graphs of average cell densities of neurons (H) activated astrocytes (I) and microglia (J) in each brain region of Cdk5 cKO1 mice and littermate controls at P30. **P < 0.01; ***P < 0.001 (t-test). Click here for file

Additional file 3: Figure S3 Evaluation of degenerative neurons and neuron size on coronal brain section of Cdk5 cKO1 mice. (A) Degenerative neurons were evaluated by amino-cupric silver stain of different areas of the brain control and Cdk5 cKO1 mice at P30. Increase number of amino-cupric silver positive cells (brown) on cerebral cortex and striatum of Cdk5 cKO1 in comparison with control mice. (B) Representative coronal sections stained with a red fluorescent Nissl stain (NeuroTraceTM530/615) to evaluated neuronal size in control and Cdk5 cKO1 mice. No difference in neuronal size was found in CA1 pyramidal neurons between Cdk5 cKO1 and control littermates at P30. Bar graphs indicate average of neuronal size of neurons from control and Cdk5 cKO1 mice. Click here for file

Additional file 4: Figure S4 PPARγ agonist pioglitazone treatment suppresses neuroinflammation and reverses neuronal loss in Cdk5 cKO1 mice at P90. (A) Survival curves for untreated and treated Cdk5 cKO1 mice from P30 to P90. Survival rate for treated mice (n = 4) is better than that of untreated mice (n = 4). (P = 0.029 in Log-rank test and P = 0.030 in Wilcoxon test). (B) Average body weights of untreated (n = 4) and treated (n = 3) Cdk5 cKO1 male mice and littermate male Cdk5f/f (control) mice (n = 4) at P90. *P < 0.05; **P < 0.01 (t-test). (C, D) IHC cortex sections of the control, untreated, and treated Cdk5 cKO1 mice o (C) and hippocampus (D) at P90. Coronal sections from these mice were stained with antibodies for NeuN, GFAP and Iba1. Scale bar = 100 μm. (E-G) Bar graphs indicate average cell densities of neurons (E), activated astrocytes (F) and microglia cells (G) in control, untreated and treated Cdk5 cKO1 mice at P90. *P < 0.05; **P < 0.01; ***P < 0.001 between control versus Cdk5 cKO1. †P < 0.05; ‡P < 0.01; §P < 0.001 between untreated versus treated. Click here for file

Additional file 5: Movie S1 Partial improvement in abnormal limb retraction and tremors in Cdk5 conditional knockout mice treated with Pioglitazone. Click here for file
