# Supplementary figures and images for: Suppression of neuroinflammation in forebrain-specific Cdk5 conditional knockout mice by PPARγ agonist improves neuronal loss and early lethality
Source: J Neuroinflammation. 2014 Feb 5;11:28. doi: 10.1186/1742-2094-11-28 (PMC3931315; doi:10.1186/1742-2094-11-28)

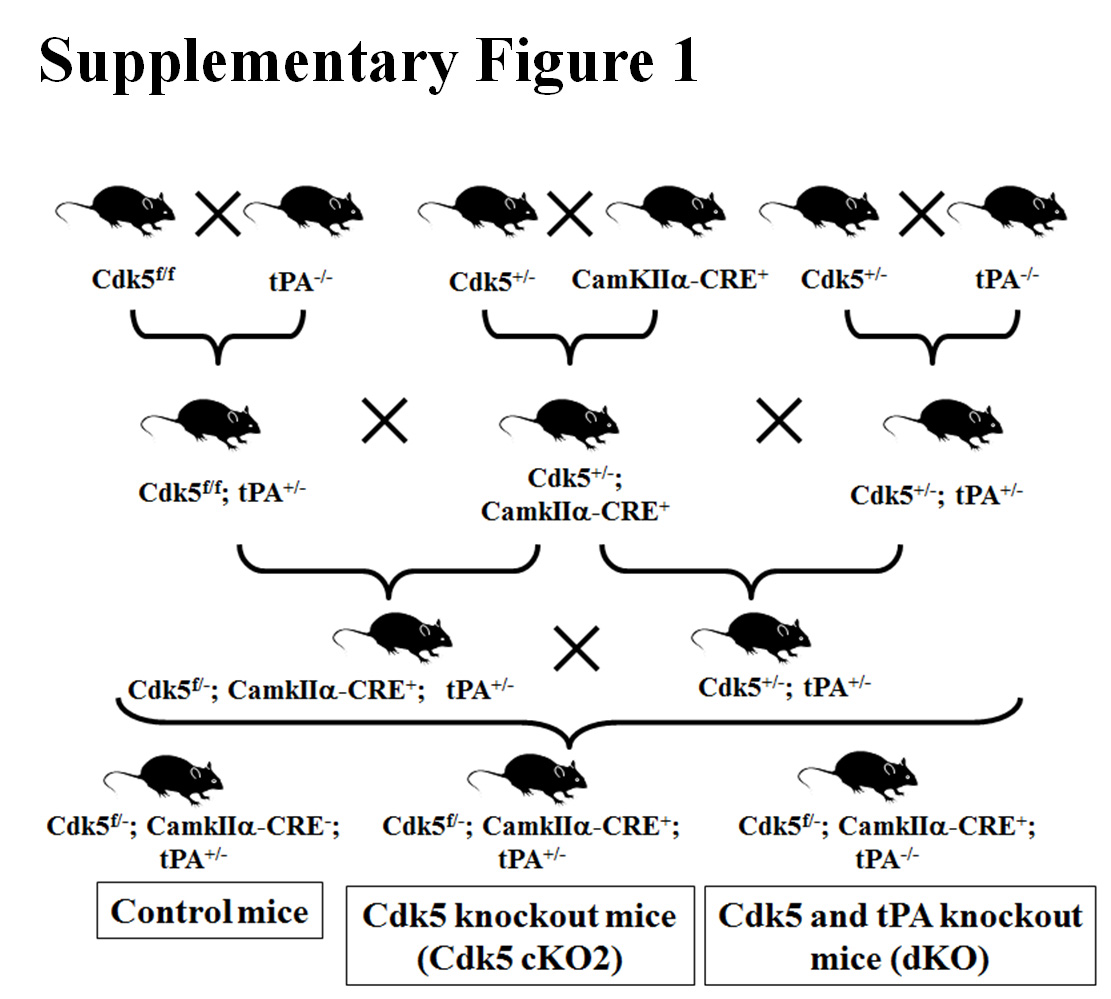

Supplement: Additional file 1: Figure S1 — Generation of Cdk5 cKO2, tPA KO mice. Cdk5 floxed (Cdk5f/f) mice were crossed with tPA knockout mice (tPA KO) to generate Cdk5f/f; tPA+/- mice. In addition, Cdk5+/- mice [7] were crossed with either CamKIIα-Cre+ [17] and tPA KO mice to generate Cdk5+/-; CamKIIα-Cre+ and Cdk5+/-; tPA KO mice, respectively. Lately, Cdk5+/-; CamKIIα-Cre+ mice were crossed either with Cdk5f/f; tPA+/- and Cdk5+/-; tPA KO mice to generate Cdk5f/-; CaMKIIα-Cre+; tPA+/- and Cdk5+/-; CamKIIα-Cre+; tPA+/-. Finally, these mice were crossed to generate Cdk5f/-; CamKIIα-Cre-; tPA+/- (control mice), Cdk5f/-; CamKIIα-Cre+; tPA+/- (Cdk5 cKO2 mice) and Cdk5f/-; CamKIIα-Cre+; tPA-/- (dKO mice). [file 1742-2094-11-28-S1.jpeg]

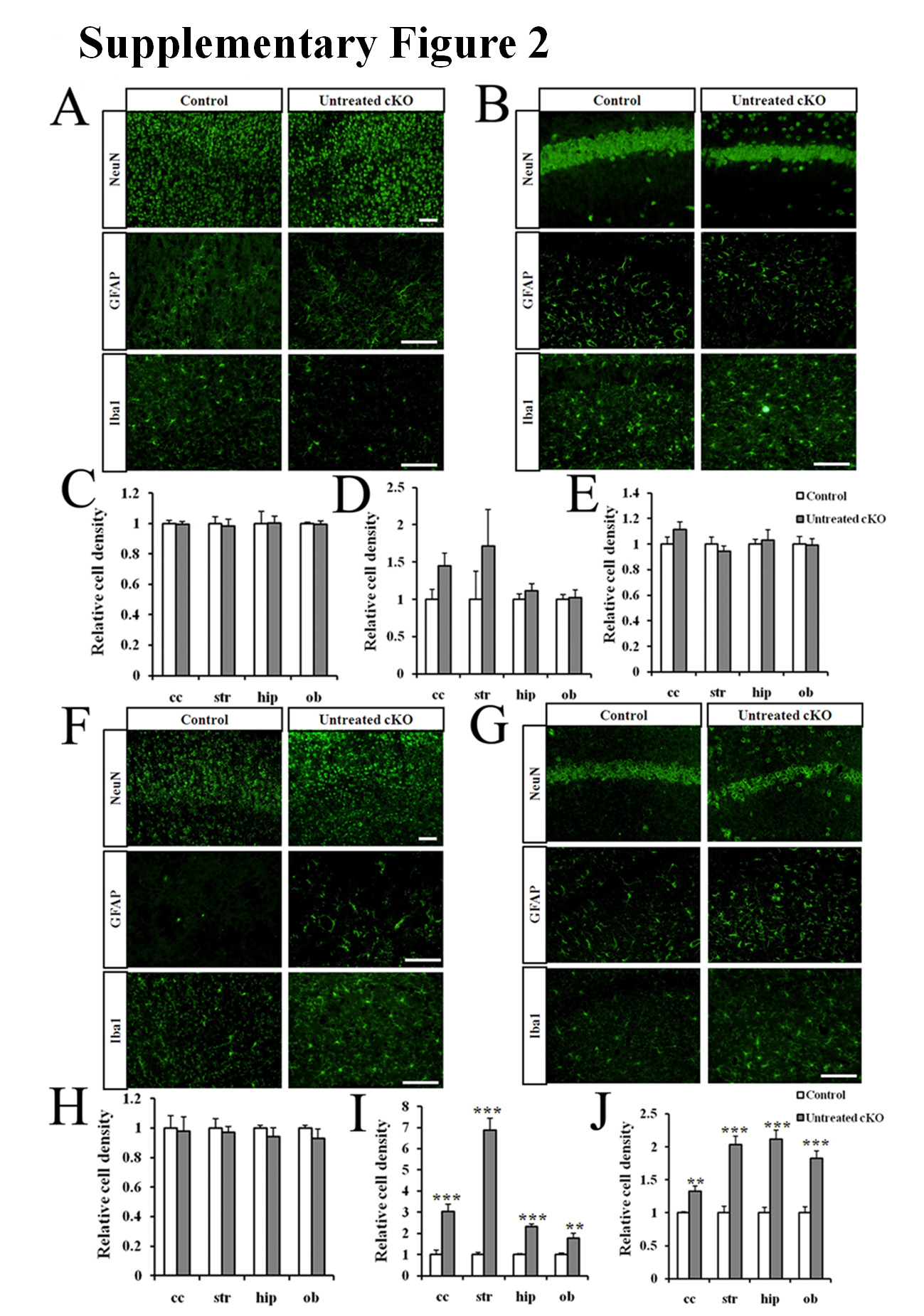

Supplement: Additional file 2: Figure S2 — Inflammation proceeds neuronal loss in Cdk5cKO mice. (A, B) IHC analysis of coronal brain sections of P21 day-old mice. Sections of cerebral cortex (A) and hippocampus (B) from control and Cdk5 cKO1 mice were stained with antibodies for NeuN, GFAP and Iba1. Scale bar = 100 μm. (C-E) Bar graphs of average cell densities of neuron (C), activated astrocytes (D) and microglia (E) in each brain region of Cdk5 cKO1 mice and littermate controls at P21. (F, G) IHC analysis of coronal brain sections of P30 day-old mice. Sections of cerebral cortex (F) and hippocampus (G) of the control and Cdk5 cKO1 mice were stained with antibodies for NeuN, GFAP and Iba1. Scale bar = 100 μm. (H-J) Bar graphs of average cell densities of neurons (H) activated astrocytes (I) and microglia (J) in each brain region of Cdk5 cKO1 mice and littermate controls at P30. **P < 0.01; ***P < 0.001 (t-test). [file 1742-2094-11-28-S2.jpeg]

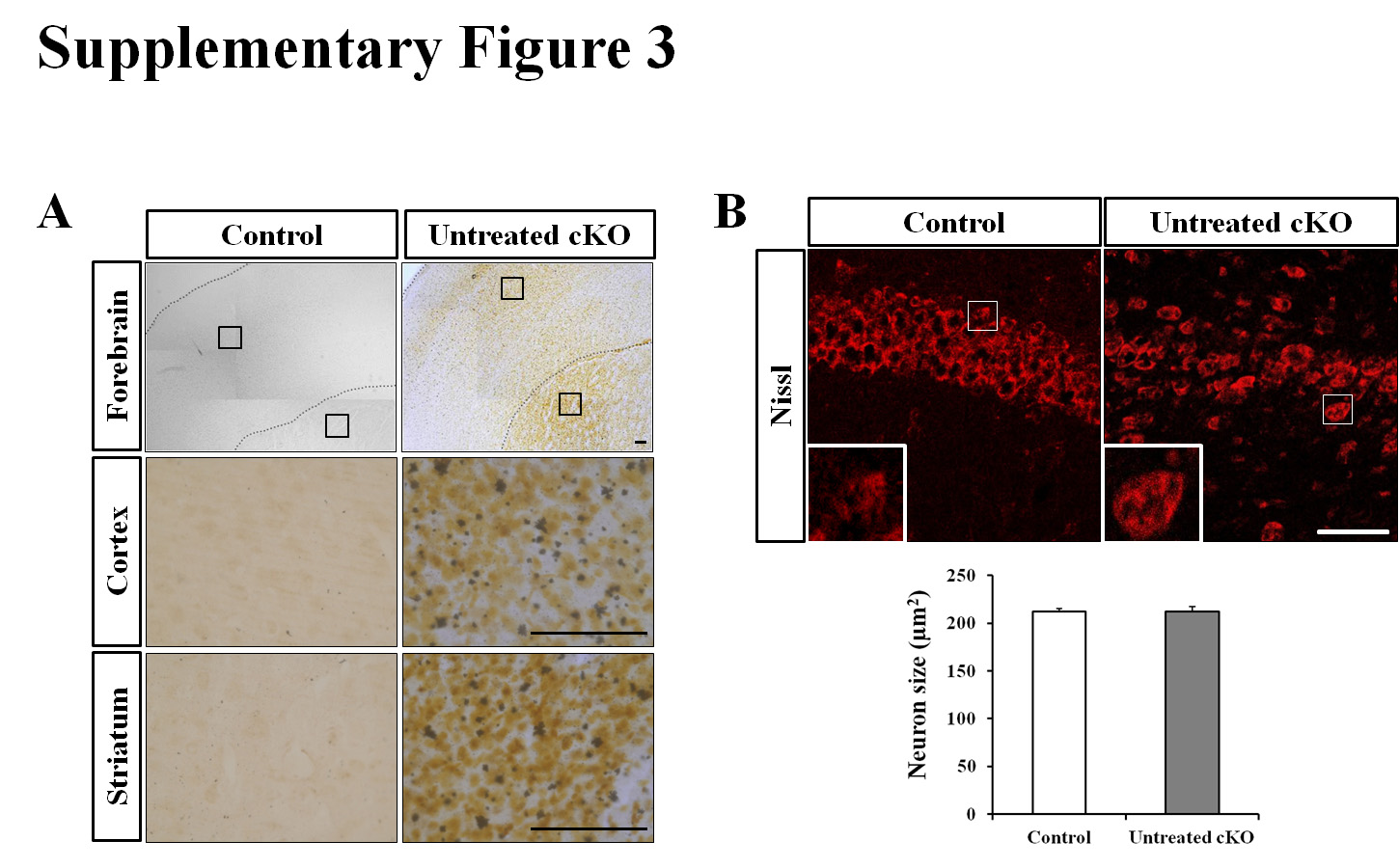

Supplement: Additional file 3: Figure S3 — Evaluation of degenerative neurons and neuron size on coronal brain section of Cdk5 cKO1 mice. (A) Degenerative neurons were evaluated by amino-cupric silver stain of different areas of the brain control and Cdk5 cKO1 mice at P30. Increase number of amino-cupric silver positive cells (brown) on cerebral cortex and striatum of Cdk5 cKO1 in comparison with control mice. (B) Representative coronal sections stained with a red fluorescent Nissl stain (NeuroTraceTM530/615) to evaluated neuronal size in control and Cdk5 cKO1 mice. No difference in neuronal size was found in CA1 pyramidal neurons between Cdk5 cKO1 and control littermates at P30. Bar graphs indicate average of neuronal size of neurons from control and Cdk5 cKO1 mice. [file 1742-2094-11-28-S3.jpeg]

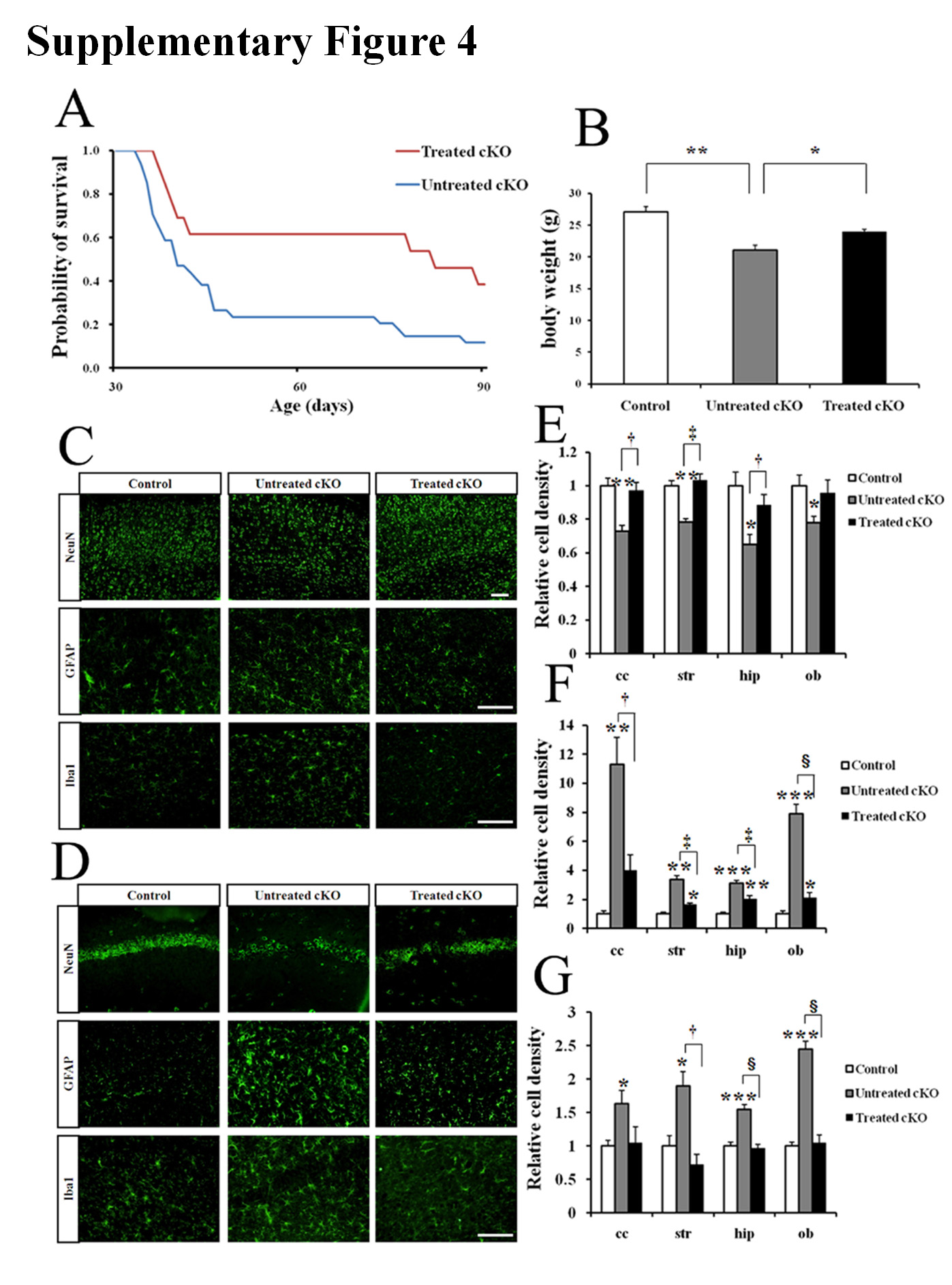

Supplement: Additional file 4: Figure S4 — PPARγ agonist pioglitazone treatment suppresses neuroinflammation and reverses neuronal loss in Cdk5 cKO1 mice at P90. (A) Survival curves for untreated and treated Cdk5 cKO1 mice from P30 to P90. Survival rate for treated mice (n = 4) is better than that of untreated mice (n = 4). (P = 0.029 in Log-rank test and P = 0.030 in Wilcoxon test). (B) Average body weights of untreated (n = 4) and treated (n = 3) Cdk5 cKO1 male mice and littermate male Cdk5f/f (control) mice (n = 4) at P90. *P < 0.05; **P < 0.01 (t-test). (C, D) IHC cortex sections of the control, untreated, and treated Cdk5 cKO1 mice o (C) and hippocampus (D) at P90. Coronal sections from these mice were stained with antibodies for NeuN, GFAP and Iba1. Scale bar = 100 μm. (E-G) Bar graphs indicate average cell densities of neurons (E), activated astrocytes (F) and microglia cells (G) in control, untreated and treated Cdk5 cKO1 mice at P90. *P < 0.05; **P < 0.01; ***P < 0.001 between control versus Cdk5 cKO1. †P < 0.05; ‡P < 0.01; §P < 0.001 between untreated versus treated. [file 1742-2094-11-28-S4.jpeg]
